# Phenomic profiling of a novel sibling species within the *Scedosporium* complex in Thailand

**DOI:** 10.1186/s12866-021-02105-5

**Published:** 2021-02-09

**Authors:** T. Kitisin, S. Ampawong, W. Muangkaew, P. Sukphopetch

**Affiliations:** 1 Department of Microbiology and Immunology, Faculty of Tropical Medicine, Mahidol University, Bangkok, Thailand; 2 Department of Tropical Pathology, Faculty of Tropical Medicine, Mahidol University, Bangkok, Thailand

**Keywords:** Phenomic profiling, *Scedosporium* species complex, Unidentified environmental isolate, Antifungal drug resistance, Scedosporiosis

## Abstract

**Background:**

*Scedosporium* species are a group of pathogenic fungi, which can be found worldwide around high human-impacted areas. Infections of *Scedosporium* have been reported in several immunocompromised and immunocompetent patients with a high mortality rate. Recently, we have isolated and identified several *Scedosporium* strains during an environmental survey in Thailand.

**Results:**

We describe the isolate, TMMI-012, possibly a new species isolated from soils in the Chatuchak public park, Bangkok, Thailand. TMMI-012 is phylogenetically related to the *Scedosporium* genus and is a sibling to *S. boydii* but shows distinct morphological and pathological characteristics. It is fast growing and highly resistant to antifungal drugs and abiotic stresses*.* Pathological studies of in vitro and in vivo models confirm its high virulence and pathogenicity.

**Conclusion:**

TMMI-012 is considered a putative novel *Scedosporium* species. The high antifungal resistance of TMMI-012 compared with its sibling, *Scedosporium* species is likely related to its clinical impact on human health.

## Background

*Scedosporium* species, including *Lomentospora prolificans* (formerly known as *Scedosporium prolificans*) are emerging filamentous fungi with septate hyphae. *Scedosporium* species have been isolated around high human-impacted areas, such as public parks and industrial sites [1]. Recently, several members of *Scedosporium* are increasingly recognized as the second most opportunistic fungi after *Aspergillus* species [2]. *Scedosporium* species can be found worldwide and cause a broad range of clinical manifestations in immunocompromised and immunocompetent patients, from localized infections to systemic mycoses, especially in the skin, lungs, and brain [3–8]. In Thailand, *S. boydii* has been found in brain tissue of renal transplant patient [9]. *S. apiospermum* has been discovered in brain abscesses of near-drowning victims during the incident of a tsunami disaster and renal transplant patients [10–12]. Most notably, patients with brain abscess and disseminated disease from *Scedosporium* infections are often at higher risk [13].

Prompt and accurate identification of the pathogen at the species level is essential due to the species-specific differences in virulence and pathogenicity [14]. Infections from *Scedosporium* are often clinically indistinguishable from other invasive/systemic mycoses, i.e., *Aspergillus* and *Fusarium* [1, 15]. However, conventional diagnostic methods from clinical specimens, including histopathological study, serological test, and fungal culture, are unspecific, unavailable commercially, or insensitive [1, 6]. Inappropriate or delayed treatment of systemic *Scedosporium* infections often increases the likelihood of patient mortality, as members of the *Scedosporium* are highly resistant to all current antifungal drugs [7, 16–18]. Therefore, precise species identification is essential to determine virulence and antifungal susceptibility between these fungal species [19]. Several molecular-based methods are being developed and used for species identification in the laboratory. Nucleotide sequencing involving several genetic loci, such as *β*-tubulin (*BT2*), calmodulin (*CAL*), the second-largest subunit of RNA polymerase II (*RPB2*), and internal transcribed spacer (*ITS*) is the global standard for precise identification at the species level within the *Scedosporium* genus [20–22]. Moreover, multi-locus sequence typing (MLST) (available online at http://mlst.mycologylab.org) is being used for genotyping and evolutionary study of *Scedosporium* species [23]. To date, after molecular phylogenetics and the “One Fungus = One Name” movement, members of the *Scedosporium* currently consist of 10 species as follows: *S. apiospermum* complex (*S. angustum*, *S. apiospermum*, *S. boydii*, *S. ellipsoideum*, and *S. fusoideum*), *S. dehoogii*, *S. minutisporum*, *S. aurantiacum, S. cereisporum*, and *S. desertorum* [13, 24]. However, the nomenclature of these *Scedosporium* species will continue to evolve, as there is a high degree of genetic variation, especially within *S. apiospermum*, *S. boydii,* and *S. dehoogii* [24].

Several cases of deep-seated *S. apiospermum* species complex infections reported in Thailand have led to our first report on environmental survey regarding the species complex diversity of *S. apiospermum* in the urban area of Bangkok and other six geographic regions in Thailand [21–23]. Besides the strains of *S. apiospermum* species complex (*S. apiospermum, S. boydii,* and *S. augustum*) found, interestingly enough, we reported unidentified *Scedosporium* isolates by genetic variation analysis [21, 22]. However, the description of those unidentified *Scedosporium* isolates as separate species requires further extensive studies, including genotyping data to support morphological, immunopathological, and clinically relevant evidence. Here we continue our study to identify a novel *Scedosporium* isolate. We describe the isolate, TMMI-012, possibly a new species isolated from soils in the Chatuchak public park, Bangkok, Thailand. TMMI-012 is phylogenetically related to the *Scedosporium* genus and is a sibling to *S. boydii* but shows distinct morphological and pathological characteristics. TMMI-012 is fast growing, highly resistant to antifungal drugs, and abiotic stresses.

Additionally, biofilm formation and chitin synthase expression, which pertains to its pathogenicity and virulence, are remarkably higher than in *S. boydii*. Infection of TMMI-012 causes the death of *Galleria mellonella* and mice infection models as well as induces strong apoptosis in innate immune cells*.* In mice, TMMI-012 infection causes more severe pulmonary and cerebral injuries with cerebral edema than *S. boydii* infection. Altogether, our study nominated a possible new species of *Scedosporium* with high virulence and pathogenicity in Thailand.

## Results

### TMMI-012 isolates a probable new sibling species to *Scedosporium boydii*

**Fig. 1:**
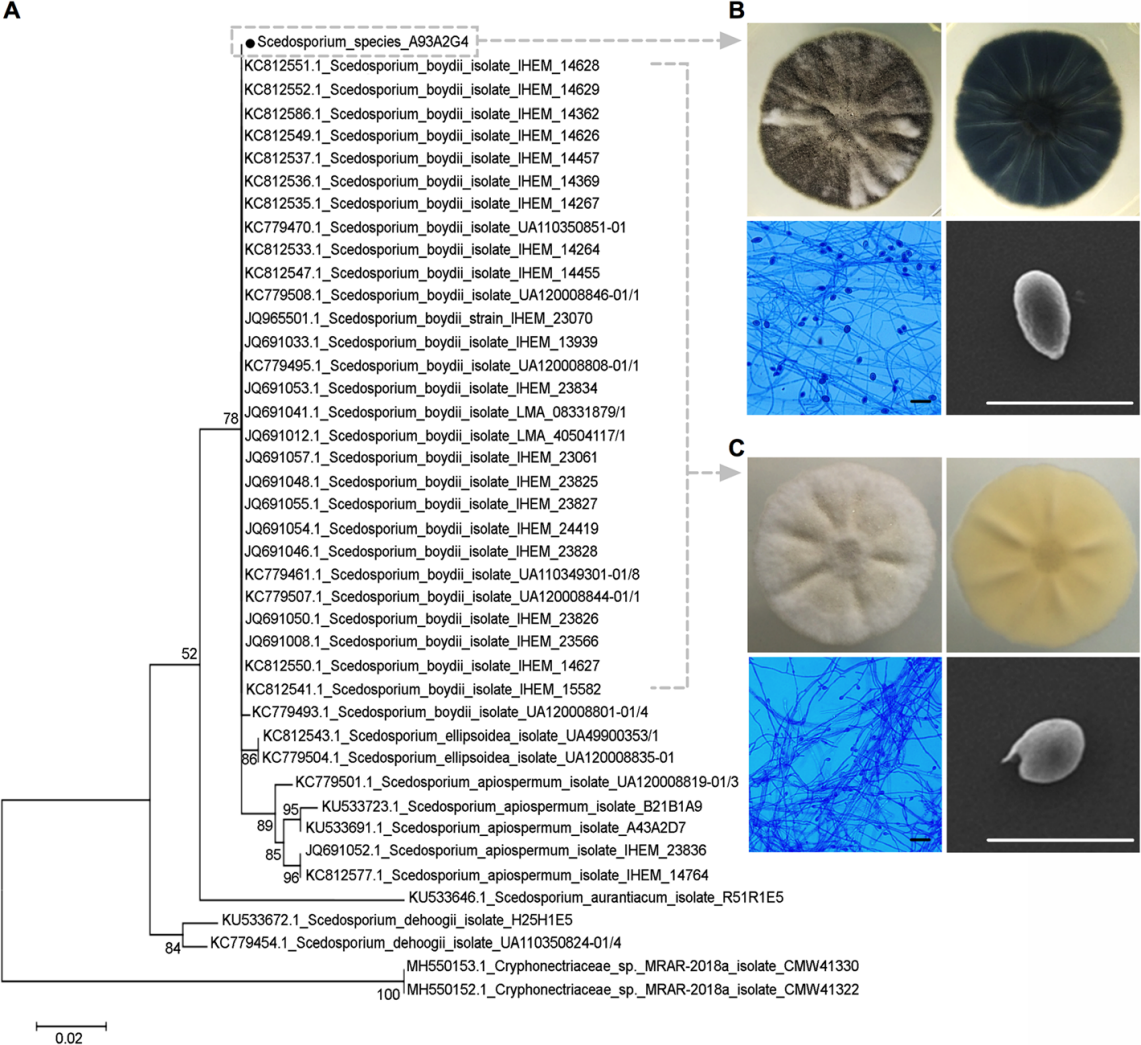
TMMI-012 identifies as a closely related *Scedosporium* to *Scedosporium boydii.* **a** Maximum-likelihood tree of β-tubulin gene sequences of TMMI-012 isolate (isolation number A93A2G4) and reference strains. The tree is drawn to scale and the branch lengths corresponding to the number of substitutions per site. Bootstrap support values above ≥50% (1000 replicates) are indicated at the branches. Accession numbers of *Scedosporium* sequence and known strains are retrieved from GenBank. *Cryphonectriaceae* sp. MRAR-2018a isolates: CMW41330 and CMW41322 were used as outgroups. **b** Morphological features of TMMI-012 (top-view: upper left, reverse-view: upper right) 7-day-old culture at 37 °C on PDA. Microscopical examination with lactophenol cotton blue stain of conidia (lower left, scale bar ~ 20 μm) and the SEM micrograph of conidium (lower right, scale bar ~ 10 μm). Notice the conidial size is 8.18 ± 0.16 μm. (*n* = 30). **c** Morphological features of *S. boydii* (top-view: upper left, reverse-view: upper right) 7-day-old culture at 37 °C on PDA. Microscopical examination with lactophenol cotton blue stain of conidia (lower left, scale bar ~ 20 μm) and the SEM micrograph of conidium (lower right, scale bar ~ 10 μm). Notice the conidial size is 4.08 ± 0.23 μm. (*n* = 30)

Since our reports on the environmental survey of *S. apiospermum* species complex in Bangkok [21, 23] and other six geographic regions in Thailand [22], we have collected and identified *Scedosporium* isolates using MLST sequencing technique in which sequences are amplified at eight genetic loci: actin (*ACT*), *CAL*, *RPB2*, manganese superoxide dismutase (*SOD2*), *ITS*, transcription elongation factor 1α (*TEF-1α*), *TUB*, and *BT2* [21–23]. Our previous results revealed 18 isolates that cannot be linked to the known specific *Scedosporium* species; thus they potentially severed as a new species. Interestingly, by stress sensitivity assay, we preliminary found that an unidentified *Scedosporium* isolate, TMMI-012 (*Scedosporium* isolate-A93A2G4, GenBank-KX382895.1), showed the highest resistance to abiotic stresses than other 17 isolates (data not shown). Therefore, we chose TMMI-012 for phylogenetic, morphological, and pathological studies. To construct the phylogeny, we performed PCR (polymerase chain reaction) amplification of the *β*-tubulin (*TUB*) gene exons 5 and 6 for TMMI-012 and obtained a successful single band of approximately 650 bp on gel after electrophoresis. The generated *β*-tubulin nucleotide sequence was used to determine the sequence similarity in the NCBI database using the basic local alignment search tool (BLASTn) algorithm. The outputs were sorted based on maximum identity. The sequence-based identities with a cutoff of ≥97% were considered significant. We aligned the *β*-tubulin sequence from TMMI-012 and related species from GenBank. After regions of sequences with ambiguous alignments and gaps were excluded, the final data sets of 42 sequences were used to generate the tree shown in Fig. 1a. In this tree, TMMI-012 was not assigned to any known *Scedosporium* species but placed in the same cluster of *S. boydii* as supported by bootstrap value [BV] = 78%. Moreover, TMMI-012 was in the same subclade as *S. apiospermum* (BV = 89%)*.* These subclades, including *S. aurantiacum* formed a clade (BV = 52%), which is sister to a supported (BV = 84%) clade comprising of *S. dehoogii*.

The TMMI-012 isolate was clearly identified by the presence of a characteristic *Scedosporium* anamorph. Although, TMMI-012 was in the same cluster of *S. boydii*, however*,* these two fungi showed distinct morphologies. TMMI-012 colony showed dark gray to black at the top view and dark olive green at the reverse view. Microscopic examination of TMMI-012 revealed, septate hyphae, solitary conidiophores on aerial mycelium, smooth-wall, cylindrical, producing pale brown, ovoid, or ellipsoidal conidia. The colony proliferated at 37 °C on potato dextrose agar (PDA) with a conidial size of 8.18 ± 0.16 μm measured by scanning electron microscopy (SEM) (Fig. 1b). Whereas *S. boydii* CBS 120157 showed white cottony colonies in both top and reverse views. Microscopic examination of *S. boydii* CBS 120157 revealed septate hyphae with simple long or short conidiophores that bore single conidium or in a small group. The conidia were unicellular and oval with a larger end toward the apex. The colony proliferated at 37 °C on PDA with a conidial size of 4.08 ± 0.23 μm measured by SEM (Fig. 1c). Therefore, we proposed that TMMI-012 isolate is a probable new sibling species of *S. boydii.*

### TMMI-012 isolates exhibits higher growth kinetics and stress resistance than *S. boydii*

**Fig. 2:**
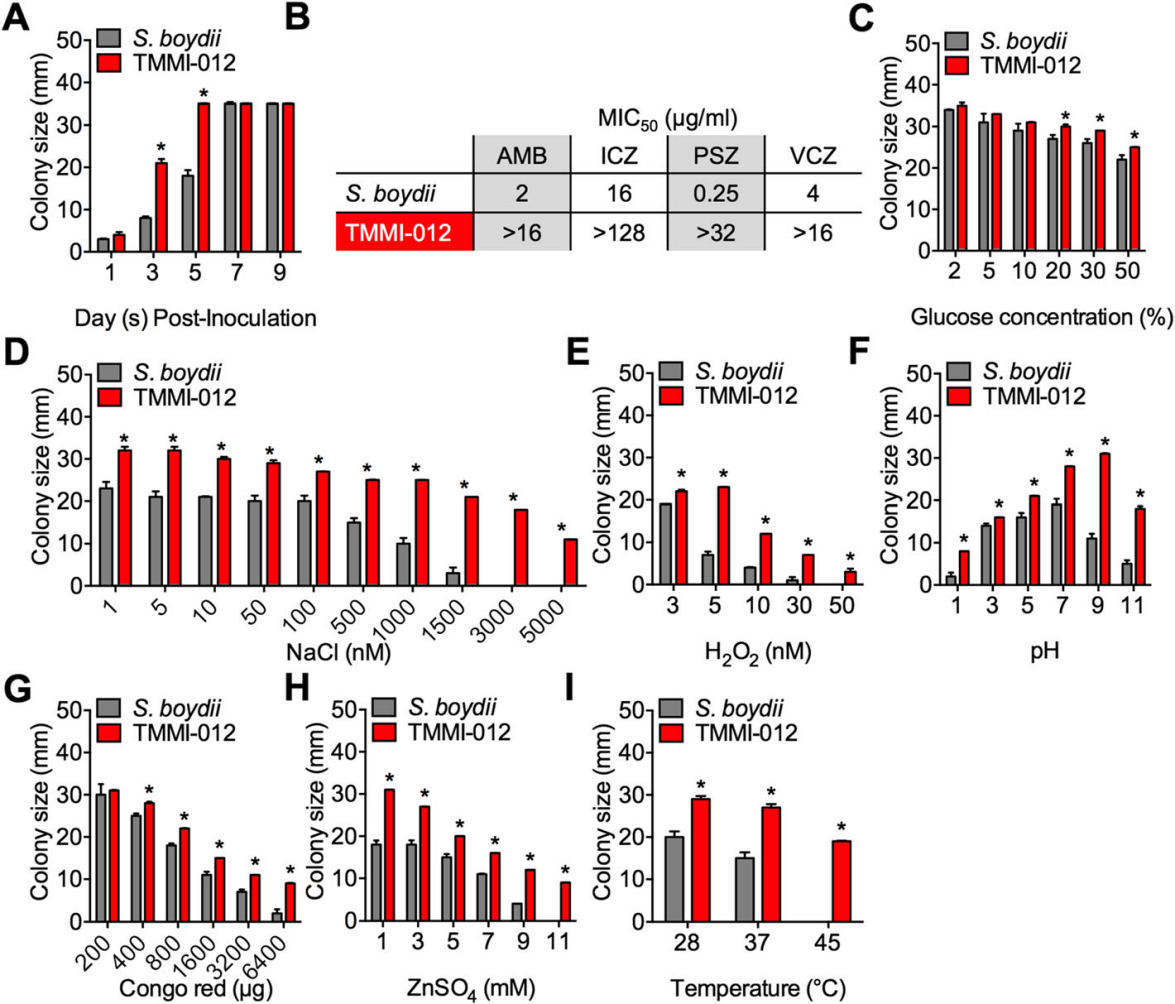
TMMI-012 exhibits higher growth kinetics and stresses resistance than *S. boydii.* **a** Growth kinetics of fungal cultures on SDA at 37 °C for 9 days. **b** Table showing minimal inhibitory concentration (MIC_50_, μg/ml) of various anti-fungal drugs. Abbreviations are as follows: AMB, amphotericin B; ICZ, itraconazole; PSZ, posaconazole; VCZ, voriconazole. Responses to glucose (**c**), NaCl (**d**), H_2_O_2_ (**e**), pH (**f**), Congo red (**g**), ZnSO_4_ (**h**), and temperature (**i**) of *S. boydii* and TMMI-012 shown in various culture conditions. Data shown are from *S. boydii* and TMMI-012 in various culture conditions (*n* = 3) and shown as mean ± SD; *P*-value calculated by one-way ANOVA with Tukey’s post hoc test; *P < 0.05

To determine the growth kinetics of TMMI-012, the diameter of colonies was measured in comparison with *S. boydii* CBS 120157. Fungal spores (1 × 10^6^ conidia/mL) were prepared and used at 1 × 10^4^ conidia/well, counted using a hematocytometer, and serial dilutions from 10^− 1^–10^− 6^ were prepared using normal saline. The fungal diameter was measured using a vernier caliper. Moreover, in both isolates, no growth was observed at higher dilutions (10^− 5^ and 10^− 6^). Our results revealed that the TMMI-012 isolate grew remarkably faster in D1 and D3 after inoculation than *S. boydii* CBS 120157 (Fig. 2a). Moreover, the growth rate of TMMI-012 is similar to that observed *S. apiospermum* species complex isolates (*S. apiospermum*, *S. boydii*, and *S. augusta*) (data not shown). We determined the antifungal drug susceptibility of TMMI-012 by minimum inhibitory concentration (MIC) of antifungal drugs, such as amphotericin B (AMB), itraconazole (ICZ), posaconazole (PSZ), and voriconazole (VCZ). The results revealed that most antifungal drugs showed MIC_50_ range of 0.25 –> 128 μg/mL. Interestingly, TMMI-012 showed more resistance to all tested antifungal drugs than *S. boydii* (Fig. 2b), which showed the highest resistance to ICZ (MIC_50_, > 128 μg/mL) and PSZ (MIC_50_, > 32 μg/mL). However, *S. boydii* showed the highest resistance to ICZ (MIC_50_, > 16 μg/mL) and VCZ (MIC_50_, 4 μg/mL) but not PSZ (MIC_50_, 0.25 μg/mL). The results suggested that the variation in response to antifungals exists even in closely related species. Thus, our results revealed that the antifungal drug resistance of TMMI-012 isolates might contribute to its pathogenicity in humans.

To determine the stress sensitivity of TMMI-012, fungal growth under various stress conditions was determined and compared with *S. boydii* CBS 120157. For osmotic stress, the colony diameter of TMMI-012 isolate was remarkably higher than *S. boydii* by 20% of glucose concentration or higher (Fig. 2c). Moreover, TMMI-012 isolate was remarkably less sensitive to NaCl and grew in high NaCl concentrations of approximately 5000 mM when compared with *S. boydii* (Fig. 2d). For oxidative stress, TMMI-012 isolate was remarkably less sensitive to H_2_O_2_, and grew in concentrations of approximately 50 mM (Fig. 2e), whereas *S. boydii* was moderately sensitive to H_2_O_2_ and grew in concentrations of approximately 30 mM. Furthermore, TMMI-012 isolate was remarkably less sensitive to all H_2_O_2_ concentrations than *S. boydii*. For pH stress, *S. boydii* was moderately resistant to pH environments, but the maximum fungal colony diameter was observed at pH 7. However, the colony diameter of the TMMI-012 isolate was remarkably higher than *S. boydii* at all pH conditions and grew best at pH 9 (Fig. 2f). For cell wall stress, Congo red was used to probe the fungal cell wall’s construction and stress response [25]. The results revealed that Congo red concentration-dependently inhibits the growth of both isolates. However, TMMI-012 isolate had a colony diameter of approximately 6400 μg/mL of Congo red, which was remarkably higher than *S. boydii* (Fig. 2g). For Zinc stress, *S. boydii* showed slight resistance to ZnSO_4_ with a concentration of approximately 9 mM. Moreover, TMMI-012 was remarkably resistant and grew in a concentration of approximately 11-mM ZnSO_4_ (Fig. 2h). For thermal stress, *S. boydii* was more sensitive to heat stress and grew at a temperature of approximately 37 °C. However, the colony diameter of TMMI-012 isolate was remarkably higher than *S. boydii*, which showed heat intolerance at approximately 45 °C (Fig. 2i). In this regard, we also compared the stress sensitivity of TMMI-012 with other related *Scedosporium* species (including *S. apiospermum* and *S. augusta*). It was revealed that TMMI-012 was remarkably resistant to stresses than *S. apiospermum* and *S. augusta* (data not shown). Therefore, these findings suggest that TMMI-012 has a strong ability to adapt to various abiotic stresses, thus contributing to its virulence.

### TMMI-012 induces monocyte death in vitro and shortens lifespan in vivo

**Fig. 3:**
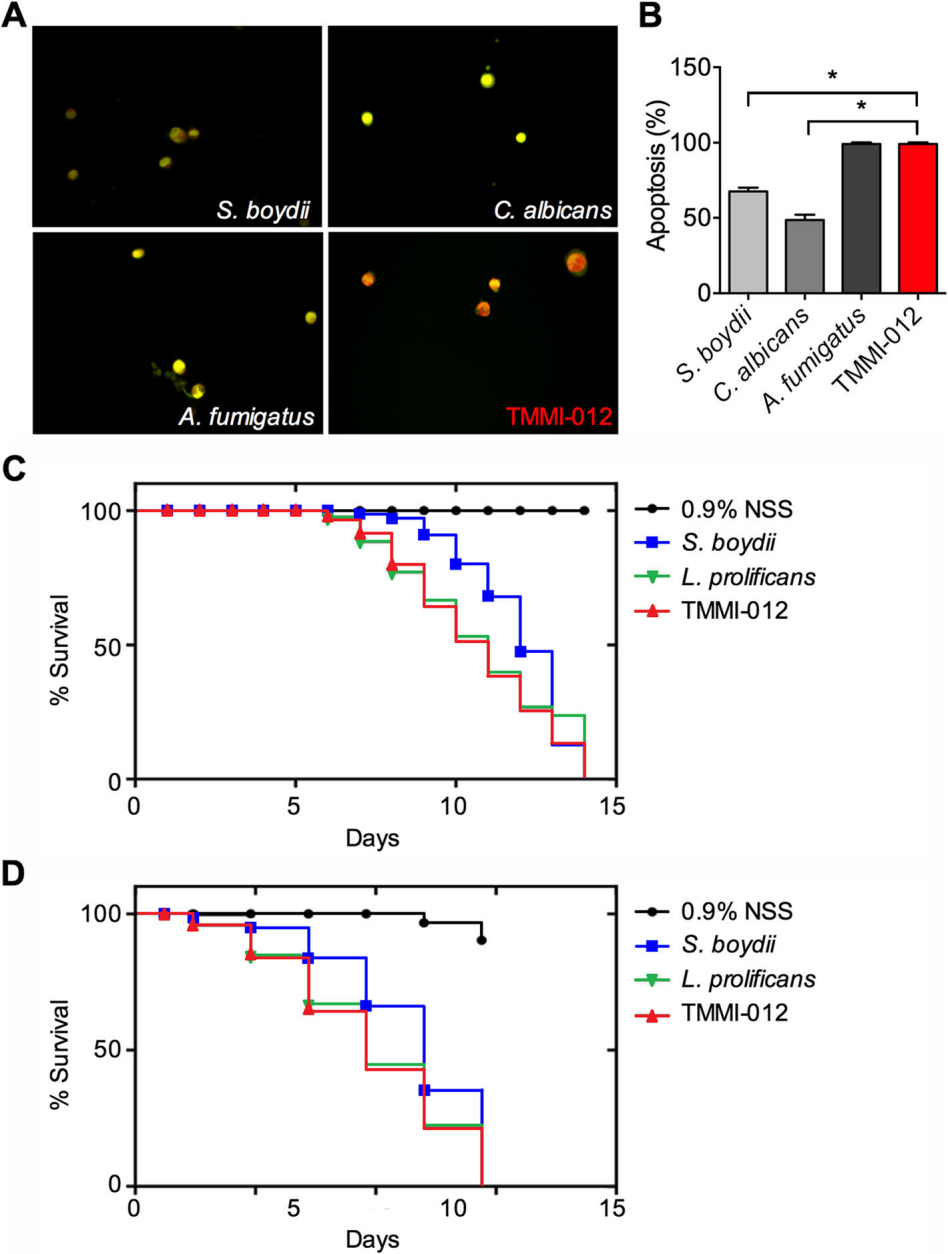
TMMI-012 induces monocyte death in vitro and shortens lifespan in vivo. **a** Acridine orange-ethidium bromide (AO/EB) staining of human monocyte cell line (THP-1) after being infected with *S. boydii*, *C. albicans*, *A. fumigatus*, or TMMI-012. **b** Percentage of cell death by apoptosis of THP-1 cells after being infected with *S. boydii*, *C. albicans*, *A. fumigatus*, or TMMI-012. The results are based on the analysis of apoptosis cells following AO/EB staining (*n* = 5). **c** Survival percentages during post-inoculation periods of mice infected with *S. boydii*, *L. prolificans*, TMMI-012, or uninfected controls (*n* = 3). **d** Survival percentages during post-inoculation periods of *G. mellonella* infected with *S. boydii*, *L. prolificans*, TMMI-012, or uninfected controls. Data shown as mean ± SD; *P*-value calculated by one-way ANOVA with Tukey’s post hoc test; *P < 0.05. For survival analysis, lifespan is analyzed using Kaplan-Meier analysis

To determine whether TMMI-012 impacts innate immunity, we co-cultured THP-1 cells with TMMI-012 isolate or other important opportunistic fungi, [24] including *S. boydii* CBS 120157, *C. albicans* 90,028, and *A. fumigatus* AF293, then examined the cell apoptosis using acridine orange/ethidium bromide (AO/EB) assay. The results revealed that THP-1 cells infected with each fungal isolate reduced cell viability (Fig. 3a). Apoptosis similar to *A. fumigatus* but higher than *S. boydii* and *C. albicans* was remarkably induced by TMMI-012 (Fig. 3b). Thus, these findings suggest that TMMI-012 impacts innate immunity causing apoptosis similar to *A. fumigatus.*

Previously, *S. apiospermum* species complex and *L. prolificans* have been increasingly reported in immunocompromised patients [8, 26]. Thus, to determine the pathogenicity of TMMI-012 in vivo, we determined the survival of experimented animals after being infected with TMMI-012, *S. boydii*, or *L. prolificans*. Mice (8-week-old) were constitutively recorded after post-inoculation. After inoculation with TMMI-012 or *L. prolificans* for 2–6 d, we observed severe neurological symptoms, moribund, and death in mice. Moreover, they all died within 14 d after inoculation (Fig. 3c). Similar neurological symptoms were found in *S. boydii*-infected mice but appeared later than TMMI-012 or *L. prolificans.* We also compared the survival rate of *G. mellonella* larvae infected with these fungi. In correlation to the mice, *G. mellonella* was introduced as an alternative host model for fungal infection due to their similarity to mammals with respect to their innate immune response [27]. Following the above results, *G. mellonella* larvae injected with TMMI-012 or *L. prolificans* died by the second day after inoculation. Mortality increased thereafter, and the survival rate progressively fell to 0% on day 6 (Fig. 3d). However, the mortality rate was remarkably reduced in the *S. boydii-*infected group, and all worms died on day 11 post-inoculation. These findings suggest that TMMI-012 has strong pathogenicity and virulence than *S. boydii.*

### TMMI-012 produces biofilm and chitin synthase higher than *S. boydii*

**Fig. 4:**
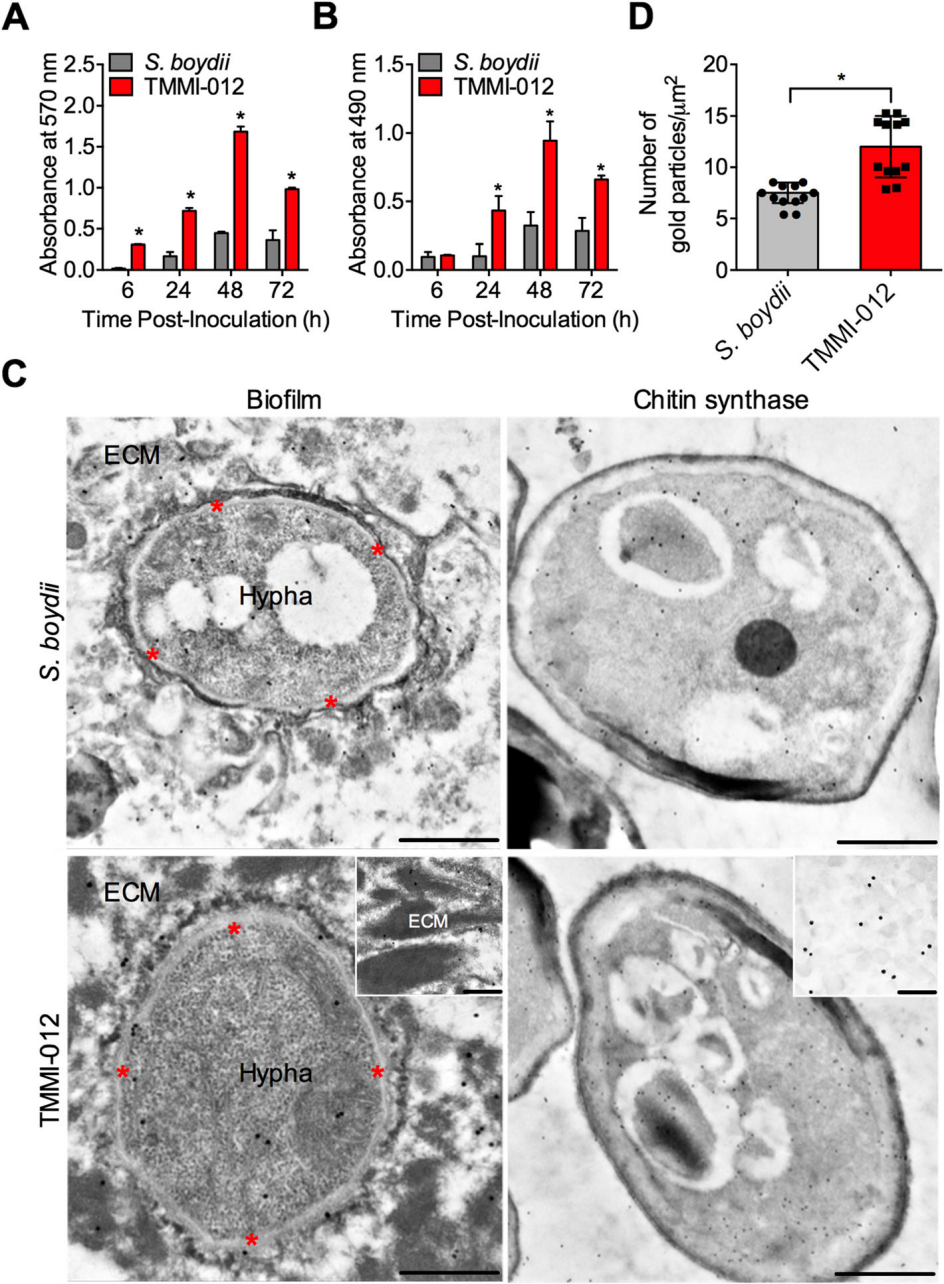
TMMI-012 produces biofilm and chitin synthase higher than *S. boydii.* Biofilms development of *S. boydii* and TMMI-012 incubated at 37 °C for 6, 24, 48, and 72 h as analyzed by crystal violet (CV) assay (**a**) and XTT assay (**b**). **c** TEM micrograph of pulmonary biofilm formation in *S. boydii* (upper left, scale bar ~ 1 μm) and TMMI-012 (lower left, scale bar ~ 500 nm) indicating the present of extracellular matrix (ECM) deposition beside the fungal hypha. Asterisk (*) indicates periplasmic space between the plasma membrane and the cell wall of fungal hypha. Inserts depict high magnification of the ECM (scale bar ~ 200 nm). TEM micrograph of immunogold localization of chitin synthase in growing conidia from *S. boydii* (upper right, scale bar ~ 1 μm) and TMMI-012 (lower right, scale bar ~ 1 μm). Inserts depict high magnification of the chitin synthase immunogold labels (scale bar ~ 200 nm). **d** Quantification of number of chitin synthase immunogold labels in growing conidia from *S. boydii* and TMMI-012. Data shown as mean ± SD (*n* = 3); *P*-value calculated by one-way ANOVA with Tukey’s post hoc test (**a**, **b**) and Student’s *t*-test (**d**); *P < 0.05

Biofilms are a complex community of microbial cells embedded in their self-secreted extracellular matrix (ECM) to protect against host immune attack and impair antifungal drug penetration [28]. Previously, a comparative study of biofilm formation by *Scedosporium* species was investigated [29]. The results showed that *Scedosporium* species could grow as biofilms in vitro, with a dense network of interconnected hyphae and ECM. Moreover, *S. boydii* produces biofilms faster than other related *Scedosporium* species [29]. To explore the biofilm formation of TMMI-012, we measured the total biofilm biomass (including ECM and cells both dead and alive) by crystal violet (CV) assays and their metabolic activity by XTT assay. The results were compared with *S. boydii.* The results revealed that TMMI-012 grows remarkably faster than *S. boydii,* especially at 6–48 h post-inoculation (Fig. 4a). At 48 h post-inoculation, the biofilm biomass was higher in TMMI-012 (mean of A570 = 1.69 ± 0.06) than *S. boydii* (0.45 ± 0.12). Interestingly, the biofilm’s metabolic activity was higher in TMMI-012 than *S. boydii* at 24–72 h (Fig. 4b)*.* At 48 h post-inoculation, the highest metabolic activity was observed in TMMI-012 (mean of A490 = 0.94 ± 0.14) compared with *S. boydii* (0.32 ± 0.09).

The ultrastructure of pulmonary biofilm formation between TMMI-012 and *S. boydii* in a mouse model of scedosporiosis was characterized by transmission electron microscopy (TEM). The formation of TMMI-012 biofilm in the pulmonary vessels was higher than *S. boydii* (Fig. 4c). Moreover, the ECM was observed at the surrounding of the fungal colony. Generally, chitin, a polysaccharide found in the cell walls of fungi, is purposed to play an important role during activation and attenuation of host immune responses [30]. Chitin is synthesized by chitin synthase enzymes and regulation of these enzymes potentially contributes to the pathogenicity of the fungus [31]. Remarkably, chitin synthase expression was revealed throughout conidial growth in both TMMI-012 and *S. boydii* by immunogold-labeling assay (Fig. 4c). However, chitin synthase expression in TMMI-012 was remarkably higher than *S. boydii* (Fig. 4d). Thus, this study revealed that TMMI-012 produces biofilm with higher expression of chitin synthase, which may promote the pathogenicity and virulence of TMMI-012.

### TMMI-012 induces severe Scedosporiosis in mice than *S. boydii*

**Fig. 5:**
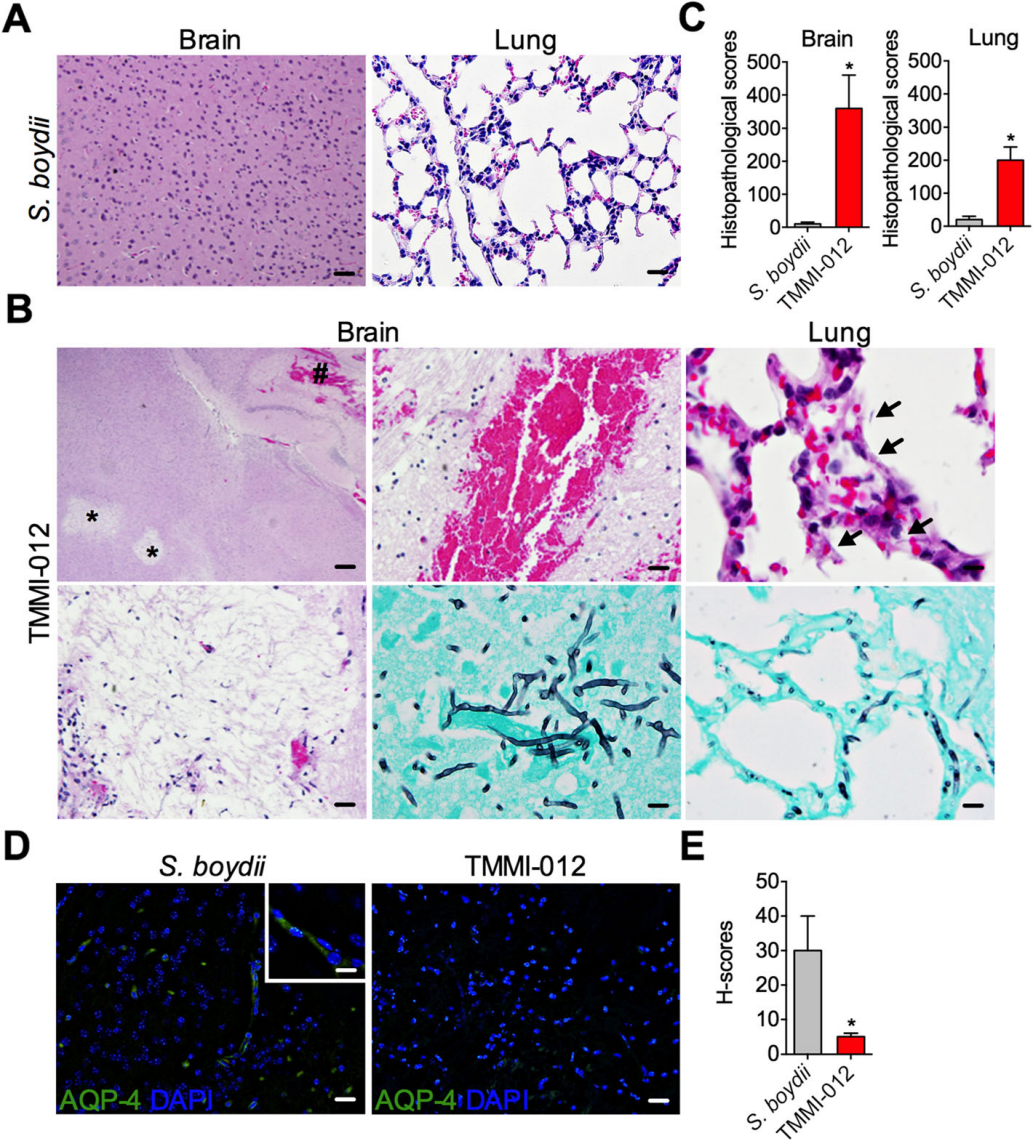
TMMI-012 induces severe scedosporiosis in mice than *S. boydii.* **a** After inoculation of *S. boydii* for a week, intact mice brain and lung are shown (scale bar ~ 20 μm). **b** After inoculation of TMMI-012 for a week, mice brain shown cerebral lesion with neuronal degradation (upper left and lower left) and hemorrhage (upper right) (scale bar ~ 20 μm). Asterisk (*) indicates neuronal degradation area; Number sign (#) indicates hemorrhage area. Mice alveolar shown alveolar septal thickening (upper). Arrow indicates accumulation of fungal hyphae. Positive stain of Grocott’s methenamine-silver is shown both in brain (lower right) and in lung tissue indicating the accumulation of fungal hyphae (scale bar ~ 10 μm). **c** Histological scores of brain and lung of mice infected with *S. boydii* and TMMI-012. **d** Representative image of immunofluorescence staining for water homeostasis marker (AQP-4) in cerebral cortex of mice infected with *S. boydii* and TMMI-012 (scale bar ~ 20 μm). Inserts depict high magnification of the AQP-4 immunofluorescence stain (scale bar ~ 10 μm). **e** Quantification of H-score of AQP-4 immunofluorescence in cerebral cortex of mice infected with *S. boydii* and TMMI-012. Data shown as mean ± SD (*n* = 3); *P*-value calculated by Student’s *t*-test; *P < 0.05

Apart from mice infected with *S. boydii*, TMMI-012-infected mice presented several histopathological changes in their brain and lungs (Fig. 5a, b). Also, neuronal degeneration with fungal hyphae deposition on the cerebral cortex and patchy hemorrhage, particularly on the hippocampus and hypothalamus, were observed in TMMI-012-infected mice (Fig. 5b). Alveolar septal thickening with cellular infiltration and the presence of fungal hyphae in the septum were also found in TMMI-012- infected mice (Fig. 5b). H-scores (histo-score) from histopathological changes in *S. boydii* and TMMI-012-infected mice (Fig. 5c), indicating that cerebral and pulmonary lesions are remarkably higher in the TMMI-012-infected group than the *S. boydii-*infected group.

Recently, we reported cerebral edema associated with abscess after *S. apiospermum* infection. We showed that brain pathology resulted from the depletion of aquaporin (AQP)-4 expression [32]. AQP-4 is a water homeostasis membrane protein that plays an essential role in regulating water movement in the brain [33]. To determine the presence of brain edema in the TMMI-012-infected group, cerebral AQP-4 was evaluated using immunofluorescence staining. The results revealed that reduced AQP-4 expression in the TMMI-012-infected mice affects the blood-brain barrier and vascular integrity (Fig. 5d, e). Therefore, our results suggest that severe cerebral edema induced by TMMI-012 infection is at least partially due to the poor AQP-4 response.

## Discussion

The nomenclature of *Scedosporium* species has been greatly revised over the last decade after using molecular phylogenetic tools, which fine-tuned the fungus even at or below the species level [24]. However, the high genetic variation among the *S. apiospermum* species complex has suggested that some intra-species variation indicates sibling species, thus should be treated as a separate species [24]. We have previously identified the environmental isolates of *Scedosporium* species by MLST [21–23]. Our studies revealed that following by stress sensitivity assay, TMMI-012 proved to be the most suitable candidate to be appointed as a new species due to its higher stress resistance than the other 17 isolates (data not shown). To further differentiate closely related *Scedosporium* species, the partial *β-*tubulin gene is needed [34]. In this study, the DNA sequence of *β*-tubulin exon 5–6 (TUB) was selected as the best broadest marker for identifying TMMI-012 within the *Scedosporium* taxa [4, 21]. We uncovered that TMMI-012 is phylogenetically related to *S. boydii* (bootstrap value [BV] = 78%) but has a distinct morphology. TMMI-012 showed the highest similarity with only a 24 bp difference in *β*-tubulin sequences compared with *S. boydii* CBS 120157 [29], which is the clinical isolate obtained from human lungs. Thus, in this study, *S. boydii* CBS 120157 was used as a reference isolate to compare the microbiological features of TMMI-012. TMMI-012 showed a bigger conidial size with a distinctive colony from *S. boydii* CBS 120157. Also, TMMI-012 grew faster than the *S. boydii* CBS 120157 isolates.

Several pathological features of the pathogenic fungi, such as resistance to environmental stresses and biofilm formation, are considered responsible for its low susceptibility to antifungal drugs [35]. To prognosticate the pathogenicity of TMMI-012, we evaluated the susceptibility of TMMI-012 to different antifungal drugs and compared the results with *S. boydii* CBS 120157. It was revealed that TMMI-012 was less susceptible to all the tested antifungal drugs than *S. boydii* CBS 120157. TMMI-012 showed resistance to more than one class of antifungal drugs, including polyenes (for AMB) and azoles (for ICZ, PSZ, and VCZ) [36], suggesting a pathogenic potential and drug resistance, therefore, may have significant impact on medical care.

Eukaryotic cells and fungi respond and adapt to different abiotic stresses in nature, which promotes their pathogenicity [37, 38]. Thus, studying fungal sensitivity under various abiotic stresses by observing its effect on growth rate can provide information on abiotic factors for fungal virulence. In this study, TMMI-012 exhibited stronger stress tolerance (to osmolarity (glucose and NaCl), oxidation (H_2_O_2_), pH, cell wall alteration (Congo red), Zinc-ion (ZnSO_4_), and temperature) than *S. boydii.* Likely, metabolic adaptation and molecular signaling pathways that activate genes involved in stress responses are involved during fungal response to abiotic stresses. Previous studies have suggested that mitogen-activated protein kinase/high-osmolarity glycerol (HOG)-1 response pathway [39], Pal/Rim pathway [40], and other related signaling pathways play an essential role in mediating stress responses in pathogenic fungi. Thus, TMMI-012 may confer tolerance to abiotic stresses via synergistic stress-resistant mechanisms.

It has been estimated that human has physically contacted a large number of fungal spores or being colonized by commensal fungi [41]. For this reason, our first line of defense as an innate system has emerged to recognize and protect the human host from pathogenic fungal invasion [42]. Considering the fungal infection of innate immune cells at the early stages of infection, we evaluated TMMI-012 infection in monocytes using the THP-1 cell line. We found that TMMI-012 induces THP-1 cell apoptosis similar to *A. fumigatus* (98–100% of cell apoptosis). Previous studies have elucidated the molecular details of the interaction between the very rapid phagocytosis and killing of *A. fumigatus* by human monocytes (within 6 h) [43, 44]. However, *A. fumigatus* also develops defense mechanisms to survive phagocytosis by producing pyomelanin and dihydroxynaphthalene (DHN) melanin [45]. These melanin, especially DHN melanin—responsible for the characteristic gray-greenish color of *A. fumigatus*—protect the fungus against various exogenous stresses, including UV-irradiation, temperatures, and reactive oxygen species. Moreover, melanin in *A. fumigatus* also plays a crucial role as a virulence factor to prevent immune recognition by masking the pathogen-associated molecular patterns on its conidial surface [46].

Nevertheless, several secondary metabolites produced from *A. fumigatus* are found to inhibit the function of neutrophils or the oxidative burst of macrophages [47, 48]. In our studies, we showed that TMMI-012 appears in a dark gray color, which may contain melanin pigments. Thus, we hypothesized that melanin pigments from TMMI-012 might promote THP-1 cellular apoptosis. In our mouse model, animals’ life span after infection with TMMI-012 or *L. prolificans* was reduced remarkably. Similar to the mouse model, TMMI-012 infection caused similar lethality in *G. mellonella* larvae. Thus, our study suggests that in vivo, the high pathogenicity of TMMI-012 is a potential risk to human health. Nevertheless, our results showed that *G. mellonella* is a valid model for TMMI-012 infections.

Previously, mechanisms of antifungal drug and abiotic stress resistance of pathogenic fungi have been well reviewed. Pathogenic fungi adapt themselves through molecular mechanisms, including biofilm formation, mitochondrial function, stress signaling, cell wall biosynthesis, pyrimidine salvage pathway, drug efflux, ergosterol biosynthesis, and lipid metabolism, which promote the multi-drug resistance, pathogenicity, and virulence of the fungi [49]. One of the critical features of pathogenic fungi is their ability to form biofilms, a network of cells embedded in their surrounding ECM, which protects against stressful environments [50]. Here, we evaluated the biofilm formation of TMMI-012 and *S. boydii* CBS 120157 isolates by CV staining to elucidate all fungal cells in the biofilm community [51]. We found that in a time-dependent manner, biofilm bulk from TMMI-012 was higher than in *S. boydii*. These results were consistent with the XTT activity assay, which revealed the metabolic activity of cells and the activity of mitochondrial dehydrogenase in living cells by evaluating the reduction of XTT to formazan [52]. Our results showed that biofilms from TMMI-012 exhibited higher XTT metabolic activity than *S. boydii* in a time-dependent manner. We also found that TMMI-012-infected mice exhibited pulmonary biofilm formation higher than in *S. boydii* infected mice*.*

Moreover, the TEM micrograph of lung samples revealed a highly dense ECM encompassing the TMMI-012 fungal hyphae than *S. boydii*. Previous studies have demonstrated a link between biofilms formation and high levels of resistance to antifungal drugs in various pathogenic fungi, including *C. albicans, Trichosporon asahii, A. fumigatus,* and *Cryptococcus neoformans* [53–56]. Thus, from our results, we suggest that the high biofilm formation of TMMI-012 confer higher levels of resistance against antifungal drugs and environmental stresses. Regulation of cell wall biosynthesis is another fungal adaptation to activate and attenuate the innate immune responses in host plants and animals [57]. Cell walls and septa of all pathogenic fungi are mainly composed of chitin, a β (1,4)-linked homopolymer of *N*-acetylglucosamine, synthesized by chitin synthases (CHSs) [58].

Moreover, disruption in chitin synthases reduces human pathogenic fungal virulence in many species, including *C. albicans* and *Exophiala* (*Wangiella*) *dermatitidis* [58–60]. In this study, we found that immunogold-labeling of chitin synthase in growing conidia of TMMI-012 was remarkably higher than in *S. boydii*. Altogether, our findings demonstrated that high levels of chitin synthase in TMMI-012 might affect the integrity of the fungal cell wall and promote fungal resistance under various stress conditions.

Here, TMMI-012-infected mice exhibited 100% lethality and died faster than the mice infected with *S. boydii* or *L. prolificans*. However, histopathological findings in the brain and lungs of TMMI-012-infected immunosuppressive mice showed more significant invasive tissue damages, including cerebral hemorrhage and edema, neuronal degradation, alveolar septal thickening, and accumulation of mononuclear cell infiltrate than in mice infected with *S. boydii*. We also revealed a more significant reduction of cerebral aquaporin (AQP)-4, a water homeostasis marker, in the TMMI-012-infected mice brain than in the *S. boydii*-infected group. A study has reported that rat perimicrovessel astrocyte foot processes express AQP-4, whereby alterations of AQP-4 cause perturbations of brain water homeostasis [61]. Moreover, highly expressed AQP-4 has been detected in the cytoplasm of mouse astrocytes [62]. A study in several types of edematous human brain tumors has also revealed the alterations of AQP-4 in reactive astrocytes [63]. Thus, AQP-4 is used as a novel marker for cerebral edema. Notably, our previous report demonstrated that *S. apiospermum* infection caused decreased expression of AQP-4 in the brain of immunosuppressive mice, proving that severe diseases, such as cerebral edema is associated with this infection.

Moreover, brain edema in *S. apiospermum*-infected mice may be due to the lack of excess water clearance caused by decreased AQP-4 [32]. Thus, our present findings demonstrate that the reduction of AQP-4 expression affects the severity of cerebral damage during TMMI-012 infection. Molecular details regarding mechanisms of pathogenesis and immune responses caused by TMMI-01—induced scedosporiosis are needed for further study.

## Conclusions

In this study, the phylogenetic and physiological characterization of TMMI-012 has presented it as a putative novel *Scedosporium* species. We demonstrated here that TMMI-012 isolate from the public park in Bangkok, Thailand exhibits stress resistance, virulence, and may expose humans to adverse health risks. The formal nomenclature of TMMI-012 is proposed to the European Confederation of Medical Mycology. Further study is planned to elucidate the distribution of TMMI-012 in clinical cases and other geographical regions. Furthermore, extensive data on molecular phylogenetics of TMMI-012 and mechanisms of antifungal drug resistance for species identification would also be highly valued.

## Materials and methods

### Fungal strains

The TMMI-012 isolate (isolation number A93A2G4; Department of Microbiology and Immunology, Faculty of Tropical Medicine, Mahidol University yeast and molds culture collection) was an unidentified isolate of *Scedosporium* sp. from our stock collection. The unidentified TMMI-012 isolate that originated from soils has previously been typed by PCR of the *β*-tubulin gene (exon 5 and 6) [21]. The standard strains *S. boydii* CBS 120157, *S. apiospermum* CBS 117410, and *Lomentospora prolificans* CM324 were obtained from el Servicio de Micología, Instituto de Salud Carlos III, Madrid, Spai*n. candida albicans* 90028 and *Aspergillus fumigatus* AF293 were obtained from the American Type Culture Collection (ATCC). Each isolate was incubated on Sabouraud dextrose agar (SDA; Difco, USA) slants at 35 °C for 7 d. Conidia were collected and suspended in phosphate-buffered saline (PBS, pH 7.4).

### Genus and species identification by DNA sequencing and analysis

DNA of TMMI-012 was extracted and purified directly from fungal colonies using an E.Z.N.A. fungal DNA mini kit (Omega Bio-tek, GA, USA). The DNA was PCR-amplified with *β*-tubulin gene-specific primers *TUB-F*: 5′-CTGTCCAACCCCTCTTACGGCGACCTG AAC-3′ and *TUB-R*: 5′-ACCCTCACCAGTATACCAATGCAAGAAAGC-3′ [20, 21]. The PCR mixture (50 μL) contained 2× GoTaq Colorless Master Mix (Promega, USA), 0.5 μM of each primer, nuclease-free water, and fungal DNA template. The amplification program was performed according to the following protocol: an initial denaturation step at 96 °C for 6 min, followed by 35 cycles of denaturation at 94 °C for 1 min, annealing at 56 °C for 1 min, and extension at 72 °C for 45 s. A final extension step at 72 °C for 10 min was done at the end of the amplification. The reaction was performed in a T100 Thermal Cycler (Bio-Rad). PCR products at a size of approximately 650 bp were purified using a FavorPrep GEL/PCR Purification Mini Kit (Favorgen Biotech Corporation, Taiwan) and sequenced using gene-specific forward and reverse primers by AITbiotech Pty Ltd. (Singapore). High-quality sequences were obtained and further edited and subjected to pairwise alignment using the BioEdit software (http://www.mbio.ncsu.edu/bioedit/bioedit.html). Edited sequences were compared with GenBank’s existing sequences using BLASTn (http://blast.ncbi.nlm.nih. gov/Blast.cgi). The generated nucleotide sequence of TMMI-012 was deposited in GenBank under accession numbers KX382895.

### Phylogenetic analysis

The sequenced data were aligned to determine β-tubulin gene variation in the TMMI-012 strain and related species downloaded from GenBank. The isolates’ evolutionary relationships were multiply aligned using the MLSTest v.1.0.1.23 software (downloaded from http://ipe.unsa.edu.ar/ software) [64]. The phylogenetic tree of all aligned sequences, excluding ambiguous alignments, gaps, and missing data, was constructed by the maximum likelihood approach based on the Tamura-Nei model in MEGA6 [65]. The phylogenetic tree was obtained by neighbor-joining analysis and drawn to scale with branch lengths, indicating the species’ evolutionary distances. A bootstrap analysis was conducted using 1000 replications.

### Morphological identification

Colony morphology of TMMI-012 was observed visually and microscopically in comparison with standard strain *S. boydii* CBS 120157. Colonies were grown on PDA (Difco, France) for macroscopic examination. They were morphologically examined using lactophenol cotton blue staining. Conidial size was measured using an image analysis program (ImageJ® v.1.36; NIH, USA). Conidia was collected by washing with sterile PBS (pH 7.2) and adjusted to a 10^5^ conidia/mL concentration for subsequent experiments.

### Scanning electron microscopy

Conidia samples from TMMI-012 and *S. boydii* CBS 120157 were primarily fixed for 1 h in 2.5% glutaraldehyde in 0.1 M sucrose phosphate buffer (SPB) at room temperature, then washed three times with SPB. Conidia samples were postfixed in 1% osmium tetroxide in SPB for 1 h and washed again. Through a series of ethanol, samples were dehydrated stepwise and air-dried overnight. Conidia samples were mounted on an aluminum stub and coated with a gold film (20 nm-thickness) using a sputter coater (Emitech K550, Ashford, UK). The ultrastructure of conidia samples was observed using an SEM (JSM-6610LV; JEOL, Japan) with a 10-kV acceleration voltage.

### Preparation of inocula

For sporulation, TMMI-012 and *S. boydii* CBS 120157 were grown on SDA at 37 °C. Mycelial tissues were used to harvest spore by suspending in normal saline with 0.03% Triton X-100. The mycelial tissues were gently vortexed and allowed to settle for 10 min at room temperature. The supernatant was processed prior to centrifugation at 3074×*g* for 5 min to obtain spores. Spores (1 × 10^6^conidia/ml) were counted using a hematocytometer, and serial dilutions from 10^− 1^–10^− 6^ were prepared in normal saline. For growth kinetics, spores were grown in SDA at 1 × 10^4^ conidia/well. The diameter of the fungal colony was measured using vernier caliper at 1, 3, 5, 7, and 9 d after inoculation.

### Antifungal susceptibility testing

For antifungal susceptibility determination, the MICs of TMMI-012 and *S. boydii* CBS 120157 were determined according to recommendations stated in the Clinical and Laboratory Standards Institute (CLSI) M38-A2 document [17]. According to the CLSI method, antifungal drugs used, including amphotericin B (AMB), itraconazole (ICZ), posaconazole (PSZ), voriconazole (VCZ) (Sigma, St. Louis, MO, USA) were obtained as reagent-grade powders. TMMI-012 and *S. boydii* CBS 120157 inocula were diluted in Gibco Roswell Park Memorial Institute (RPMI) 1640 medium, and the final inoculum in assay wells was between 0.5 × 10^3^–5 × 10^3^ CFU/mL and incubated at 35 °C for 24–48 h. According to the CLSI method, the MICs were visually determined after 24 h of incubation by selecting the lowest concentration of a drug that caused ≥50% inhibition in growth compared to drug-free controls.

### Stress sensitivity assay

The sensitivity of TMMI-012 and *S. boydii* CBS 120157 to various stresses was determined in SDA with a concentration range of stress inducers. Glucose (2–50% v/v) and sodium chloride (NaCl) (1–5000 mM) for osmotic stress induction, hydrogen peroxide (H_2_O_2_) (3–50 mM) for oxidative stress induction, media with a pH range of 1–11 for pH stress induction, Congo red (200–6400 μg) for cell wall stress induction, ZnSO_4_ (1–11 mM) for metal stress induction, and heat (28 °C, 37 °C, and 45 °C) for thermal stress induction were used. Each of the fungal spores (10 μ/well or 10^4^ conidia/well) was spotted on plates containing these stress-inducing agents. Plates were incubated at 37 °C (or otherwise stated in thermal stress), and growth was examined after 48 h of incubation. Fungal colony size (mm) was measured and compared with respect to each exposure to stress conditions.

### Human monocytic leukemia THP-1 cell culture

The human monocytic leukemia THP-1 cell line was obtained from ATCC and cultured in complete RPMI-1640 medium supplemented with 10% heat-inactivated fetal bovine serum and 1% penicillin/streptomycin (100 U/100 mg/mL). Cells were grown at a density of 10^6^ cells/mL and maintained in a 5% CO_2_ humidified incubator at 37 °C.

### Dual acridine orange/ethidium bromide fluorescent staining

THP-1 cells (10^6^ cells) were seeded with either 10^8^ CFU/mL of *S. boydii* CBS 120157, *C. albicans* 90,028, *A. fumigatus* AF293, or TMMI-012 onto 12-well plates with 2 mL of complete RPMI-1640 medium for 24 h. THP-1 cells were collected by trypsinization with 0.25% Trypsin-EDTA (ethylenediaminetetraacetic acid), washed with PBS, and centrifuged to obtain the cell pellet. The cell pellet was re-suspended in 50 μL of complete culture medium. Twenty microliters of cell suspension was mixed with 10 μL of 100 mg/mL AO/EB mixture (10-μM acridine orange and 10-μM ethidium bromide, Sigma, St. Louis, MO, USA). The apoptotic cell numbers (200 cells per sample) were immediately observed under a fluorescence microscope (Olympus/BX41).

### In vivo fungal infection analysis

BALB/cMlac mice were induced to neutropenia by intraperitoneal injection of 200-mg/kg cyclophosphamide [32]. On day 1 post-induction, leukocyte count was performed to confirm the successful neutropenic stage. To induce scedosporiosis, neutropenic mice were intravenously injected with 10^6^ conidia of TMMI-012, *S. boydii* CBS 120157, *L. prolificans* CM324, or 200 μL of 5% dextrose in 0.9% sodium chloride (D5S), which served as a mock-infected control. Clinical manifestation after injection (either death or moribund) was carefully monitored daily for 7 d. Survival analysis of mice after the infection was determined. The moribund mice were humanely euthanized with an overdose of isoflurane inhalation.

*G. mellonella* larvae were anesthetized in cooled Petri dishes (5 °C–8 °C) for a day and then injected with 5 × 10^7^ cells/mL of TMMI-012, *S. boydii* CBS 120157, *L. prolificans* CM324, or 200 μL of 5% dextrose in 0.9% sodium chloride (D5S), which served as a mock-infected control. Then, for survival analysis, the larvae were incubated in the dark without food at 37 °C.

### Biofilm formation

Biofilm formation of TMMI-012 and *S. boydii* CBS 120157 were performed by seeding 100 μL of 10^6^ cells/mL in RPMI 1640 (with L-glutamine, pH 7) (Gibco, USA) into polystyrene 96-well flat-bottomed microtiter plates at 37 °C for 6, 24, 48, and 72 h [66]. A well containing just the growth medium was used as a negative control.

### Crystal violet staining assay

Biofilm formation of TMMI-012 and *S. boydii* CBS 120157 was determined using CV staining [66]. At each indicated time point, the wells were washed twice with 200 μL of sterile PBS and air-dried for 45 min. Then, each well was stained with 100 μL of 0.5% aqueous CV solution for 5 min. The excess stain was washed off from the wells by running water and decolorized with 100 μL of 95% ethanol for 45 min. Eighty microliters of the decolorized solution in each well was collected and transferred to a new 96-well plate. Absorbance values were measured at 570 nm using a microtiter plate reader (Tecan Sunrise, Austria). The absorbance values were proportional to the biofilm biomass quality, which consists of hyphae and extracellular polymeric materials.

### XTT reduction assay

The metabolic activity of TMMI-012 and *S. boydii* CBS 120157 biofilms were accessed by adding 100-μL XTT tetrazolium salt [2,3-bis (2-methoxy-4-nitro-5-sulfophenyl)-2H-tetrazolium-5-caboxanilide/menadione] solution at each time point [66]. The plates were incubated in the dark for 2 h at 37 °C. After which, 80 μL of colored supernatant solution in each well was collected and transferred to a new microplate. The absorbance values were measured at 490 nm using a microtiter plate reader (Tecan Sunrise, Austria). The colorimetric changes in the XTT reduction assay correlated with the metabolic activity of the biofilms.

### Transmission electron microscopy

To characterize the biofilm formation between TMMI-012 and *S. boydii* CBS 120157 in vivo, cyclophosphamide-induced neutropenic BALB/c mice were infected with both fungal species as described above. Pulmonary biofilm formation was examined by TEM. Lungs were collected, couple fixed in 2.5% glutaraldehyde and 1% osmium tetroxide, dehydrated in a series of ethanol, infiltrated, and embedded in London resin-white (LR-white), sectioned into a thickness of 100 nm. The sections were stained with uranyl acetate and lead citrate and examined under a TEM (model HT7700-6610LV, Hitachi, Tokyo, Japan).

To compare the fungal virulence based on the production of chitin synthase both in TMMI-012 and *S. boydii* CBS 120157, immunogold labeling for SEM was used to determine the chitin synthase expression on their growing conidia. Two conidia strains were fixed, dehydrated, infiltrated, embedded, and then cut into thicknesses of 100 nm. The ultrathin sections were incubated in 50-mM glycine. The samples were blocked with 5% bovine serum albumin (BSA) (25,557, EMS, USA) for 10 min, incubated in rabbit polyclonal anti-chitin synthase antibody, (MyBioSource, San Diego, USA) for 1 h, incubated in goat anti-rabbit IgG conjugated with 5-nm gold particle (G7402-.4ML, Sigma Aldrich, Germany) for 1 h, and then enhanced using a silver enhancement kit (Aurion R-Gent SE-EM kit, 25,521, EMS, USA). Following the staining with uranyl acetate and lead citrate, immunolocalization was examined under TEM. Gold particles were counted on the conidia by at least 50 conidia/fungal species. The conidial area was measured using an image analysis program (ImageJ v.1.36; NIH, USA). The comparison was done using the number of gold particle/μm^2^.

### Histopathological and immunofluorescence studies

Brains and lungs from TMMI-012 and *S. boydii* CBS 120157 infection in cyclophosphamide-induced neutropenic BALB/c mice were collected, fixed, and submitted for standard tissue processing and cut into a thickness of 5 μm. The sections were stained with Grocott’s methenamine-silver, hematoxylin, and eosin to examine histopathological changes, including the presence of fungal colonies, septal thickening and congestion, cellular degeneration, hemorrhage, and granulomatous lesions between scedosporiosis mice infected with TMMI-012 and *S. boydii*. These lesions were scored using the combination of H-score (the multiplication of pathological severity; 0 = absent, 1 = mild, 2 = moderate, and 3 = severe and lesion distribution; 0 = absent, 1 = < 25% distribution, 2 = 25–50% distribution, and 3 = > 50% distribution/section). Also, to examine blood vessels’ integrity during TMMI-012- and *S. boydii*-induced scedosporiosis, immunofluorescence staining of aquaporin (AQP)-4, a cerebral water channel membrane protein, was performed. The sections were deparaffinized in xylene and heat-induced antigen retrieval was performed using citrate buffer, pH 6.0. Following incubation with the rabbit anti-AQP-4 (MyBioSource, USA) for 1 h, at room temperature, the sections were soaked with the DyLight 488 anti-rabbit IgG (VectaFlour Duet reagent, Vector, USA). Immunolocalized AQP-4 was semi-quantified using H-score (the multiplication between the distribution of positive area (0–100%) and the intensity of immunolabeling score (0 = absent, 1–3 = mild, moderate, and strong expression). The percentage area of expression was measured using ImageJ. Briefly, ten images of labeled areas were captured and transformed into binary images. Immunolocalization was defined by the threshold mode and determined as an area fraction (%).

### Statistical analysis

Experimental data were analyzed using GraphPad Prism 6 (GraphPad Software, San Diego, CA, USA). Each assay was performed in triplicate or otherwise stated. The log-rank test was used for survival time determination. Significant differences at *P* < 0.05 were calculated using nonparametric Student’s *t*-test or one-way analysis of variance with Tukey’s multiple comparison test.

## Data Availability

The generated nucleotide sequence of TMMI-012 (isolation number A93A2G4) can be accessed in GenBank under accession numbers KX382895. The datasets generated and/or analyzed during the current study are available from the corresponding author on reasonable request.

## Abbreviations

MLST: Multi-locus sequence typing
MICs: Minimum inhibitory concentrations
AMB: Amphotericin B
ICZ: Itraconazole
PSZ: Posaconazole
VCZ: Voriconazole
AO/EB: Acridine orange/ethidium bromide
CV: Crystal violet
XTT: 2,3-bis (2-methoxy-4-nitro-5-sulfophenyl)-2H-tetrazolium-5-caboxanilide/menadione
AQP-4: Aquaporin-4

## References

[1] Cortez KJ, Roilides E, Quiroz-Telles F, Meletiadis J, Antachopoulos C, Knudsen T, Buchanan W, Milanovich J, Sutton DA, Fothergill A (2008). Infections caused by Scedosporium spp. Clin Microbiol Rev.

[2] Borman AM, Palmer MD, Delhaes L, Carrère J, Favennec L, Ranque S, Gangneux JP, Horré R, Bouchara JP (2010). Lack of standardization in the procedures for mycological examination of sputum samples from CF patients: a possible cause for variations in the prevalence of filamentous fungi. Med Mycol.

[3] Giraud S, Bouchara JP (2014). Scedosporium apiospermum complex: diagnosis and species identification. Curr Fungal Infect Rep.

[4] Chen M, Zeng J, De Hoog GS, Stielow B (2016). Gerrits Van Den Ende AH, Liao W, Lackner M. the ‘species complex’ issue in clinically relevant fungi: a case study in Scedosporium apiospermum. Fungal Biol.

[5] Fisher JF, Shadomy S, Teabeaut JR, Woodward J, Michaels GE, Newman MA, White E, Cook P, Seagraves A, Yaghmai F (1982). Near-drowning complicated by brain abscess due to Petriellidium boydii. Arch Neurol.

[6] Guarro J, Kantarcioglu AS, Horré R, Rodriguez-Tudela JL, Cuenca Estrella M, Berenguer J, de Hoog GS (2006). Scedosporium apiospermum: changing clinical spectrum of a therapy-refractory opportunist. Med Mycol.

[7] Gilgado F, Cano J, Gené J, Sutton DA, Guarro J (2008). Molecular and phenotypic data supporting distinct species statuses for Scedosporium apiospermum and Pseudallescheria boydii and the proposed new species Scedosporium dehoogii. J Clin Microbiol.

[8] Luplertlop N (2018). Pseudallescheria/Scedosporium complex species: from saprobic to pathogenic fungus. J Mycol Med.

[9] Satirapoj B, Ruangkanchanasetr P, Treewatchareekorn S, Supasyndh O, Luesutthiviboon L, Supaporn T (2008). Pseudallescheria boydii brain abscess in a renal transplant recipient: first case report in Southeast Asia. Transplant Proc.

[10] Leechawengwongs M, Milindankura S, Liengudom A, Chanakul K, Viranuvatti K, Clongsusuek P (2007). Multiple Scedosporium apiospermum brain abscesses after near-drowning successfully treated with surgery and long-term voriconazole: a case report. Mycoses..

[11] Larbcharoensub N, Chongtrakool P, Wirojtananugoon C, Watcharananan SP, Sumethkul V, Boongird A, Jirasiritham S (2013). Treatment of a brain abscess caused by Scedosporium apiospermum and Phaeoacremonium parasiticum in a renal transplant recipient. Southeast Asian J Trop Med Public Health.

[12] Garzoni C, Emonet S, Legout L, Benedict R, Hoffmeyer P, Bernard L, Garbino J (2005). Atypical infections in tsunami survivors. Emerg Infect Dis.

[13] Hawksworth DL, Crous PW, Redhead SA, Reynolds DR, Samson RA, Seifert KA, Taylor JW, Wingfield MJ, Abaci O, Aime C (2011). The Amsterdam declaration on fungal nomenclature. IMA Fungus.

[14] Rougeron A, Giraud S, Alastruey-Izquierdo A, Cano-Lira J, Rainer J, Mouhajir A, Le Gal S, Nevez G, Meyer W, Bouchara JP (2018). Ecology of Scedosporium species: present knowledge and future research. Mycopathologia..

[15] Cimon B, Carrère J, Vinatier JF, Chazalette JP, Chabasse D, Bouchara JP (2000). Clinical significance of Scedosporium apiospermum in patients with cystic fibrosis. Eur J Clin Microbiol Infect Dis.

[16] Gilgado F, Serena C, Cano J, Gené J, Guarro J (2006). Antifungal susceptibilities of the species of the Pseudallescheria boydii complex. Antimicrob Agents Chemother.

[17] Lackner M, de Hoog GS, Verweij PE, Najafzadeh MJ, Curfs-Breuker I, Klaassen CH, Meis JF (2012). Species-specific antifungal susceptibility patterns of Scedosporium and Pseudallescheria species. Antimicrob Agents Chemother.

[18] Zeng J, Kamei K, Zheng Y, Nishimura K (2004). Susceptibility of Pseudallescheria boydii and Scedosporium apiospermum to new antifungal agents. Nihon Ishinkin Gakkai Zasshi.

[19] Lu Q (2011). Gerrits van den Ende AH, Bakkers JM, sun J, Lackner M, Najafzadeh MJ, Melchers WJ, li R, de Hoog GS. Identification of Pseudallescheria and Scedosporium species by three molecular methods. J Clin Microbiol.

[20] Bernhardt A, Sedlacek L, Wagner S, Schwarz C, Würstl B, Tintelnot K (2013). Multilocus sequence typing of Scedosporium apiospermum and Pseudallescheria boydii isolates from cystic fibrosis patients. J Cyst Fibros.

[21] Luplertlop N, Pumeesat P, Muangkaew W, Wongsuk T, Alastruey-Izquierdo A (2016). Environmental screening for the Scedosporium apiospermum species complex in public parks in Bangkok, Thailand. PLoS One.

[22] Luplertlop N, Muangkaew W, Pumeesat P, Suwanmanee S, Singkum P (2019). Distribution of Scedosporium species in soil from areas with high human population density and tourist popularity in six geographic regions in Thailand. PLoS One.

[23] Wongsuk T, Pumeesat P, Luplertlop N (2017). Genetic variation analysis and relationships among environmental strains of Scedosporium apiospermum sensu stricto in Bangkok, Thailand. PLoS One.

[24] Ramirez-Garcia A, Pellon A, Rementeria A, Buldain I, Barreto-Bergter E, Rollin-Pinheiro R, de Meirelles JV, Xisto MIDS, Ranque S, Havlicek V (2018). Scedosporium and Lomentospora: an updated overview of underrated opportunists. Med Mycol.

[25] Ram AF, Klis FM (2006). Identification of fungal cell wall mutants using susceptibility assays based on Calcofluor white and Congo red. Nat Protoc.

[26] Sedlacek L, Graf B, Schwarz C, Albert F, Peter S, Würstl B, Wagner S, Klotz M, Becker A, Haase G (2015). Prevalence of Scedosporium species and Lomentospora prolificans in patients with cystic fibrosis in a multicenter trial by use of a selective medium. J Cyst Fibros.

[27] Singkum P, Suwanmanee S, Pumeesat P, Luplertlop N (2019). A powerful in vivo alternative model in scientific research: Galleria mellonella. Acta Microbiol Immunol Hung.

[28] Desai JV, Mitchell AP, Andes DR (2014). Fungal biofilms, drug resistance, and recurrent infection. Cold Spring Harb Perspect Med.

[29] Rollin-Pinheiro R, de Meirelles JV, Vila TVM, Fonseca BB, Alves V, Frases S, Rozental S, Barreto-Bergter E (2017). Biofilm formation by Pseudallescheria/ Scedosporium species: a comparative study. Front Microbiol.

[30] Lenardon MD, Munro CA, Gow NA (2010). Chitin synthesis and fungal pathogenesis. Curr Opin Microbiol.

[31] Borghi E, Morace G, Borgo F, Rajendran R, Sherry L, Nile C, Ramage G (2015). New strategic insights into managing fungal biofilms. Front Microbiol.

[32] Ampawong S, Luplertlop N (2019). Experimental scedosporiosis induces cerebral oedema associated with abscess regarding Aquaporin-4 and Nrf-2 depletions. Biomed Res Int.

[33] Ng WH, Hy JW, Tan WL, Liew D, Lim T, Ang BT, Ng I (2009). Aquaporin-4 expression is increased in edematous meningiomas. J Clin Neurosci.

[34] Lackner M, de Hoog GS, Yang L, Moreno LF, Ahmed SA, Andreas F, Kaltseis J, Nagl M, Lass-Flörl C, Risslegger B (2014). Proposed nomenclature for Pseudallescheria, Scedosporium and related genera. Fungal Divers.

[35] Berman J, Krysan DJ (2020). Drug resistance and tolerance in fungi. Nat Rev Microbiol.

[36] Robbins N, Caplan T, Cowen LE (2017). Molecular evolution of antifungal drug resistance. Annu Rev Microbiol.

[37] Alonso-Monge R, Román E, Arana DM, Pla J, Nombela C (2009). Fungi sensing environmental stress. Clin Microbiol Infect.

[38] Brown AJ, Budge S, Kaloriti D, Tillmann A, Jacobsen MD, Yin Z, Ene IV, Bohovych I, Sandai D, Kastora S (2014). Stress adaptation in a pathogenic fungus. J Exp Biol.

[39] Hohmann S (2002). Osmotic stress signaling and osmoadaptation in yeasts. Microbiol Mol Biol Rev.

[40] Selvig K, Alspaugh JA (2011). pH response pathways in fungi: adapting to host-derived and environmental signals. Mycobiology..

[41] Drummond RA, Gaffen SL, Hise AG, Brown GD (2014). Innate defense against fungal pathogens. Cold Spring Harb Perspect Med.

[42] Portnoy JM, Williams PB, Barnes CS (2016). Innate immune responses to fungal allergens. Curr Allergy Asthma Rep.

[43] Marr KA, Koudadoust M, Black M, Balajee SA (2001). Early events in macrophage killing of Aspergillus fumigatus conidia: new flow cytometric viability assay. Clin Diagn Lab Immunol.

[44] Loeffler J, Haddad Z, Bonin M, Romeike N, Mezger M, Schumacher U, Kapp M, Gebhardt F, Grigoleit GU, Stevanović S (2009). Interaction analyses of human monocytes co-cultured with different forms of Aspergillus fumigatus. J Med Microbiol.

[45] Heinekamp T, Thywißen A, Macheleidt J, Keller S, Valiante V, Brakhage AA (2013). Aspergillus fumigatus melanins: interference with the host endocytosis pathway and impact on virulence. Front Microbiol.

[46] Pihet M, Vandeputte P, Tronchin G, Renier G, Saulnier P, Georgeault S, Mallet R, Chabasse D, Symoens F, Bouchara JP (2009). Melanin is an essential component for the integrity of the cell wall of Aspergillus fumigatus conidia. BMC Microbiol.

[47] Mitchell CG, Slight J, Donaldson K (1997). Diffusible component from the spore surface of the fungus Aspergillus fumigatus which inhibits the macrophage oxidative burst is distinct from gliotoxin and other hyphal toxins. Thorax..

[48] Fallon JP, Reeves EP, Kavanagh K (2010). Inhibition of neutrophil function following exposure to the Aspergillus fumigatus toxin fumagillin. J Med Microbiol.

[49] Hokken MWJ, Zwaan BJ, Melchers WJG, Verweij PE (2019). Facilitators of adaptation and antifungal resistance mechanisms in clinically relevant fungi. Fungal Genet Biol.

[50] Ramage G, Rajendran R, Sherry L, Williams C (2012). Fungal biofilm resistance. Int J Microbiol.

[51] Melo AS, Bizerra FC, Freymüller E, Arthington-Skaggs BA, Colombo AL (2011). Biofilm production and evaluation of antifungal susceptibility amongst clinical Candida spp. isolates, including strains of the Candida parapsilosis complex. Med Mycol.

[52] Jin Y, Yip HK, Samaranayake YH, Yau JY, Samaranayake LP (2003). Biofilm-forming ability of Candida albicans is unlikely to contribute to high levels of oral yeast carriage in cases of human immunodeficiency virus infection. J Clin Microbiol.

[53] Chandra J, Kuhn DM, Mukherjee PK, Hoyer LL, McCormick T, Ghannoum MA (2001). Biofilm formation by the fungal pathogen Candida albicans: development, architecture, and drug resistance. J Bacteriol.

[54] Di Bonaventura G, Pompilio A, Picciani C, Iezzi M, D’Antonio D, Piccolomini R (2006). Biofilm formation by the emerging fungal pathogen Trichosporon asahii: development, architecture, and antifungal resistance. Antimicrob Agents Chemother.

[55] Kaur S, Singh S (2014). Biofilm formation by Aspergillus fumigatus. Med Mycol.

[56] Martinez LR, Casadevall A (2015). Biofilm formation by Cryptococcus neoformans. Microbiol Spectr.

[57] Henar Valdivieso M, Durán A, Roncero C (1999). Chitin synthases in yeast and fungi. EXS..

[58] Munro CA, Gow NA (2001). Chitin synthesis in human pathogenic fungi. Med Mycol..

[59] Munro CA, Winter K, Buchan A, Henry K, Becker JM, Brown AJ (2001). Chs1 of Candida albicans is an essential chitin synthase required for synthesis of the septum and for cell integrity. Mol Microbiol.

[60] Liu H, Kauffman S, Becker JM, Szaniszlo PJ (2004). Wangiella (Exophiala) dermatitidis WdChs5p, a class V chitin synthase, is essential for sustained cell growth at temperature of infection. Eukaryot Cell.

[61] Rash JE, Yasumura T, Hudson CS, Agre P, Nielsen S (1998). Direct immunogold labeling of aquaporin-4 in square arrays of astrocyte and ependymocyte plasma membranes in rat brain and spinal cord. Proc Natl Acad Sci U S A.

[62] Nicchia GP, Frigeri A, Liuzzi GM, Santacroce MP, Nico B, Procino G, Quondamatteo F, Herken R, Roncali L, Svelto M (2000). Aquaporin-4-containing astrocytes sustain a temperature- and mercury-insensitive swelling in vitro. Glia..

[63] Saadoun S, Papadopoulos MC, Davies DC, Krishna S, Bell BA (2002). Aquaporin-4 expression is increased in oedematous human brain tumours. J Neurol Neurosurg Psychiatry.

[64] Tomasini N, Lauthier JJ, Llewellyn MS, Diosque P (2013). MLSTest: novel software for multi-locus sequence data analysis in eukaryotic organisms. Infect Genet Evol.

[65] Tamura K, Stecher G, Peterson D, Filipski A, Kumar S (2013). MEGA6: molecular evolutionary genetics analysis version 6.0. Mol Biol Evol.

[66] Pumeesat P, Muangkaew W, Ampawong S, Luplertlop N (2017). Candida albicans biofilm development under increased temperature. New Microbiol.

